# Automatic segmentation of bladder cancer on MRI using a convolutional neural network and reproducibility of radiomics features: a two-center study

**DOI:** 10.1038/s41598-023-27883-y

**Published:** 2023-01-12

**Authors:** Yusaku Moribata, Yasuhisa Kurata, Mizuho Nishio, Aki Kido, Satoshi Otani, Yuki Himoto, Naoko Nishio, Akihiro Furuta, Hiroyuki Onishi, Kimihiko Masui, Takashi Kobayashi, Yuji Nakamoto

**Affiliations:** 1 Department of Diagnostic Imaging and Nuclear Medicine, Graduate School of Medicine, Kyoto University, 54 Shogoin Kawahara-cho, Sakyo-ku, Kyoto, 606-8507 Japan; 2 Preemptive Medicine and Lifestyle-Related Disease Research Center, Kyoto University Hospital, Kyoto, Japan; 3 Department of Diagnostic Radiology, Shiga General Hospital, Moriyama, Japan; 4 Department of Radiology, Osaka Red Cross Hospital, Osaka, Japan; 5 Department of Urology, Osaka Red Cross Hospital, Osaka, Japan; 6 Department of Urology, Kyoto University Graduate School of Medicine, Kyoto, Japan

**Keywords:** Machine learning, Image processing, Bladder

## Abstract

This study aimed to develop a versatile automatic segmentation model of bladder cancer (BC) on MRI using a convolutional neural network and investigate the robustness of radiomics features automatically extracted from apparent diffusion coefficient (ADC) maps. This two-center retrospective study used multi-vendor MR units and included 170 patients with BC, of whom 140 were assigned to training datasets for the modified U-net model with five-fold cross-validation and 30 to test datasets for assessment of segmentation performance and reproducibility of automatically extracted radiomics features. For model input data, diffusion-weighted images with b = 0 and 1000 s/mm^2^, ADC maps, and multi-sequence images (b0-b1000-ADC maps) were used. Segmentation accuracy was compared between ours and existing models. The reproducibility of radiomics features on ADC maps was evaluated using intraclass correlation coefficient. The model with multi-sequence images achieved the highest Dice similarity coefficient (DSC) with five-fold cross-validation (mean DSC = 0.83 and 0.79 for the training and validation datasets, respectively). The median (interquartile range) DSC of the test dataset model was 0.81 (0.70–0.88). Radiomics features extracted from manually and automatically segmented BC exhibited good reproducibility. Thus, our U-net model performed highly accurate segmentation of BC, and radiomics features extracted from the automatic segmentation results exhibited high reproducibility.

## Introduction

Bladder cancer (BC) is the tenth most common malignancy worldwide, with an increasing mortality rate^1^. There are various treatment options for BC depending on the clinical staging, including transurethral resection of bladder tumor (TURBT), intravesical therapy, total cystectomy, radiation therapy, chemotherapy, and immunotherapy^2^. Although TURBT provides pathological information on the local staging of BC, the quality of TURBT depends on the expertise of the urologist. Consequently, MRI is now an essential tool for the preoperative local staging of BC, including the recently released Vesical Imaging Reporting and Data System (VI-RADS)^3–5^.

Medical image analysis using radiomics approaches has garnered increasing attention in recent years. Radiomics extracts and analyzes large amounts of advanced quantitative imaging features with the goal of objective diagnostic imaging. Several studies have employed radiomics to predict BC staging, including muscle invasion^6–10^. For this type of research, tumor segmentation is necessary; however, manual segmentation is labor-intensive, time-consuming, and lacks objectivity. Thus, highly accurate automatic tumor segmentation is necessary to create regions of interest (ROIs) with less effort and conduct radiomics studies on a large scale.

Various methods for BC segmentation have been reported. Segmentation with convolutional neural network (CNN)-based methods has recently received growing attention^11–18^. U-net is a model for automatic segmentation that was initially developed for biomedical science and has emerged as a popular method of CNN-based segmentation of medical images^19^. Several studies of automated segmentation of BC on MRI using CNN-based methods have been reported. However, these studies were conducted at a single institution with a small number of cases and, therefore, lacked sufficient generalizability for clinical application^14–18^. To overcome these limitations, it is necessary to conduct research with a larger number of MR images of patients with BC obtained using multi-vendor MR scanners at multiple institutes. Furthermore, the robustness of radiomics features extracted using automatic segmentation is important for performing texture analysis with automatically acquired ROIs.

This study thus aimed to conduct a modified U-Net automatic segmentation of BC with high generalization performance on two-center, multi-vendor MR images using a CNN, an approach that has never been attempted before. In addition, we investigated the reproducibility of radiomics features extracted from manually and automatically segmented BC.

## Materials and methods

This two-center retrospective study was approved, and the need for written informed consent was waived, because of the retrospective study design, by the Ethics Committee of Kyoto University Graduate School and Faculty of Medicine and the Medical Ethics Committee of Osaka Red Cross Hospital (R2695 and J-0202, respectively). All methods were conducted in accordance with the Declaration of Helsinki.

### Patients

**Figure 1:**
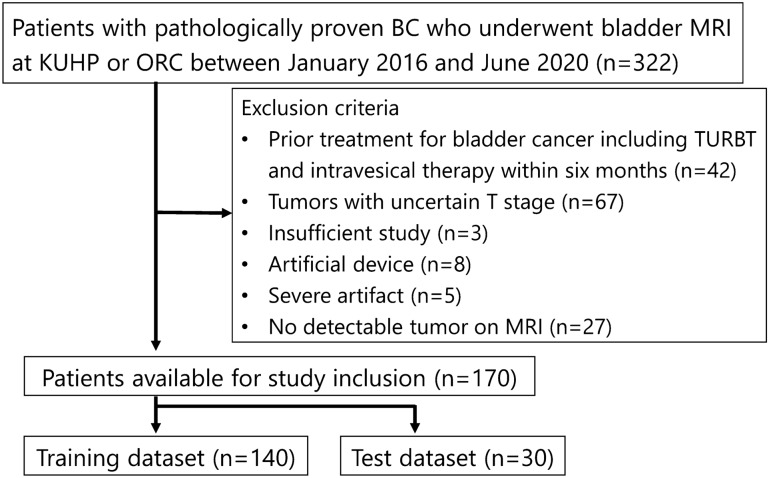
Flow chart of patient selection. *KUHP* Kyoto University Hospital, *ORC* Osaka Red Cross Hospital, *TURBT* transurethral resection of bladder tumor.

This study included consecutive patients with pathologically proven BC who underwent preoperative bladder MRI at Kyoto University Hospital and Osaka Red Cross Hospital between January 2016 and June 2020. Pathological diagnosis of BC was confirmed by TURBT or cystectomy. Exclusion criteria were (1) prior treatment for BC within 6 months, including TURBT and intravesical therapy, (2) tumors with uncertain T stage according to the European Association of Urology guidelines^20^, (3) insufficient MR images, (4) artificial devices, (5) severe artifacts, and (6) no detectable tumor on MRI. A final total of 170 patients enrolled in this study were randomly divided into two datasets: a training dataset of 140 patients and a test dataset of 30 patients (Fig. 1). Two board-certified radiologists (Y.M. and N.N. with 12 and 10 years of experience in urogenital radiology, respectively) searched the clinical and pathological records for age, sex, histological grade, and the presence of muscle invasion. The presence of muscle invasion was determined based on the European Association of Urology guidelines^20^.

### MRI protocol

MR images were obtained using a 1.5-T or 3-T scanner with a phased-array coil. The following MR scanners were used: Skyra, Prisma, Avanto (Siemens Healthineers), Achieva, Ingenia, Intera (Philips Healthcare), and SIGNA EXCITE (GE Healthcare). At a minimum, each study included axial T2-weighted images (T2WIs), axial T1-weighted images (T1WIs), and axial diffusion-weighted images (DWIs). The b-values applied to DWI were as follows: b = 0, 1000 s/mm^2^; b = 0, 500, 1000 s/mm^2^; and b = 0, 100, 500, 1000 s/mm^2^. Apparent diffusion coefficient (ADC) maps were automatically constructed with a mono-exponential decay model, using all b-values. Axial DWIs with b = 0 and 1000 s/mm^2^ and axial ADC maps were used as input sources for subsequent analyses. The MR scanners and their corresponding number of patients and DWI parameters are shown in Supplementary Table. The matrix size of DWI and ADC maps was 110–192 × 80–128.

### Image annotation and processing

A board-certified radiologist with 12 years of experience in urogenital radiology (Y.M.) manually segmented BCs on each slice of axial DWIs with b = 1000 s/mm^2^ using 3D Slicer (https://www.slicer.org) with reference to MR images of other sequences and pathological reports. Another board-certified radiologist with 12 years of experience (Y.K.) verified the ROIs for all cases. The labeled ROIs were regarded as the reference standard for tumor segmentation.

Axial DWIs were resized to 128 × 128 pixels. MR signal intensities (SIs) were normalized according to the following Eq. (1):

1 $$normalized\_SI = \frac{SI - mean\_SI}{{12 \times SD\_SI}}$$

where normalized_SI, mean_SI, and SD_SI denote the normalized SI, mean SI of the images, and standard deviation (SD) of the SI of the images, respectively.

### U-net architecture

**Figure 2:**
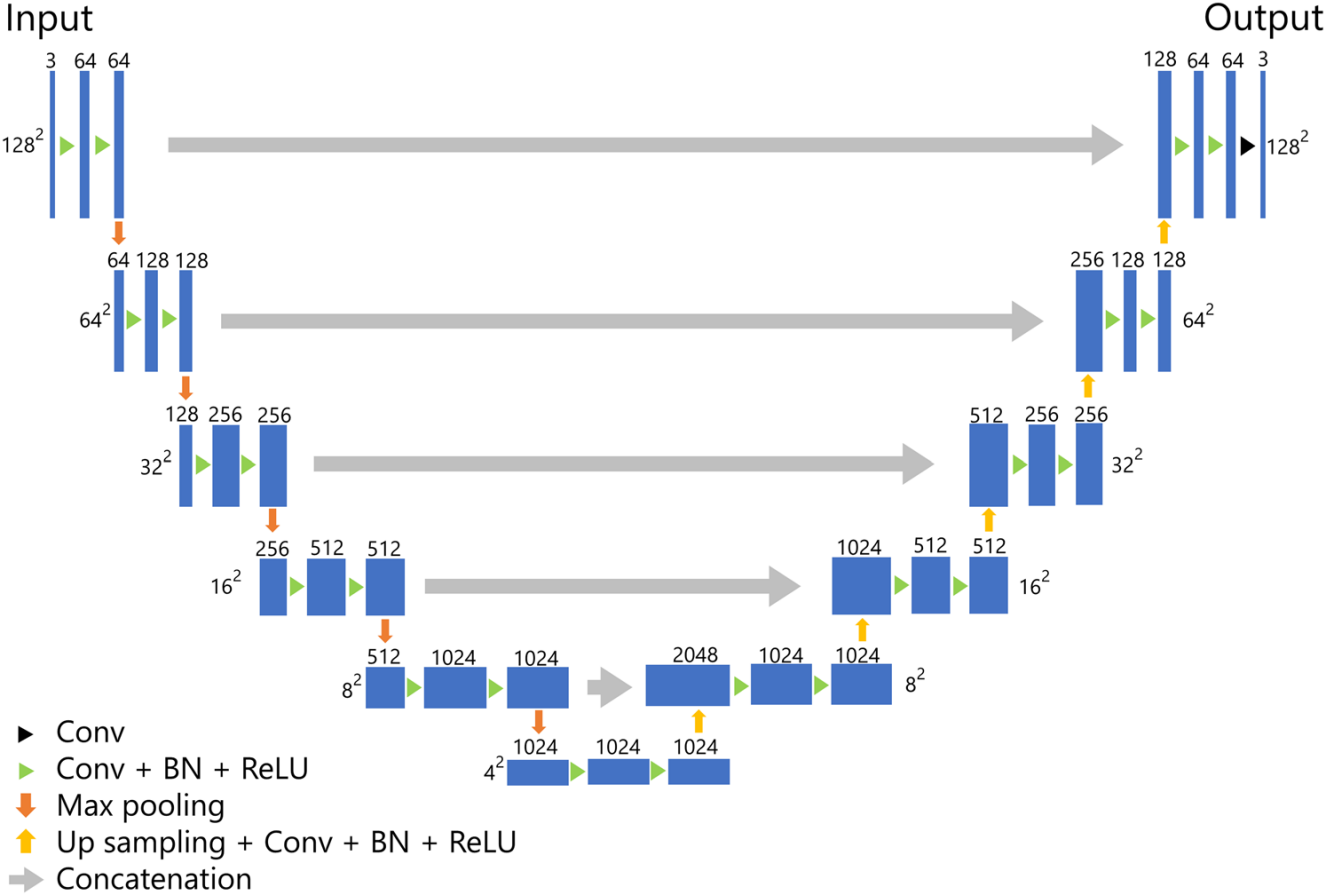
U-Net architecture. *Conv* convolution, *BN* batch normalization, *ReLU* rectified linear unit.

The architecture of our U-net-based model for the segmentation of BC is presented in Fig. 2. Our model was composed of five layers, which was deeper than the original U-net composed of four layers. For training the model, we employed the Adam optimizer as the optimizer with the cost function of Dice loss. The epochs, batch size, and initial learning were set to 30, 56, and 0.001, respectively. During the training, we performed five-fold cross-validation using 80% of the patients for training and 20% for validation.

To evaluate the impact of input image type on training effectiveness, we used single-sequence images (b0 images, b1000 images, or ADC maps) and multi-sequence images (b0 images + b1000 images + ADC maps) as input data and compared their segmentation performance. When using single-sequence images, one of the b0 images, b1000 images, or ADC maps was input to all of the triple channels. When using multi-sequences, the three sequence images were input to each of the triple channels. The input data type with the highest accuracy in the training dataset was evaluated using the test dataset. In addition, we compared the segmentation performance of our U-Net model with previously reported segmentation networks, such as original U-Net, attention U-Net, UNet++, U^2^-Net, and TransUNet^19,21–24^.

### Segmentation accuracy

Segmentation accuracy was evaluated with the metric of Dice similarity coefficient (DSC), which was defined using Eq. (2), as follows:

2 $$DSC = \frac{2|T \cap P|}{{|T| + |P|}}$$

where |T| and |P| represent the count of voxels for the true manual segmentation (reference standard) and predicted segmentation results, respectively, and |*T* ∩ *P*| denotes the count of voxels for which BC is accurately segmented (true positive).

### Model training

Our U-net model was built and trained with Tensorflow (version 2.5.0) on a Linux workstation (Ubuntu version 18.04) with an NVIDIA GeForce RTX3090 graphics processing unit with 24 GB memory.

### Performance evaluation of the test dataset

**Figure 3:**
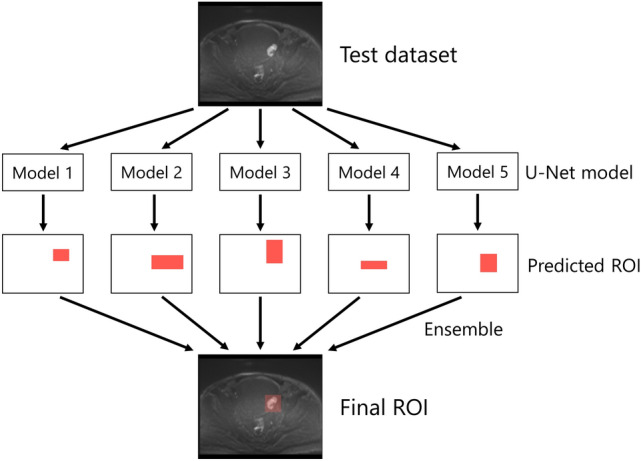
The ensemble model to predict the area of bladder cancer in the test dataset. *ROI* region of interest.

After building our U-net models using the training dataset, segmentation performance was evaluated using the test dataset. For the segmentation of BC in the test dataset, we employed an ensemble model composed of the five models created during the cross-validation process. The area predicted as BC in three or more of the five models was defined as the predicted BC. The ensemble method to predict the BC on the test dataset is presented in Fig. 3.

### Extraction of radiomics features

To evaluate the reproducibility of the radiomics features extracted from ADC maps, we compared the features calculated from manually and automatically segmented BC by our U-net model. The radiomics features were calculated using PyRadiomics (version 3.0.1). Basically, we used the default setting of PyRadiomics except for binwidth (= 0.005) and voxel array shift (= 1). The calculated radiomics features were first-order features (n = 18), shape-based features (n = 14), and high-order features (n = 75). High-order features included the following: gray-level co-occurrence matrix (GLCM, n = 24), gray-level run length matrix (GLRLM, n = 16), gray-level size zone matrix (GLSZM, n = 16), neighboring gray tone difference matrix (NGTDM, n = 5), and gray-level dependence matrix (GLDM, n = 14). The details of the radiomics features are available online for reference (https://pyradiomics.readthedocs.io/en/latest/index.html).

### Statistical analysis

Clinical characteristics of the patients in the training and test datasets were analyzed using JMP Pro (version 15.2.0, SAS Institute Inc.). Age was compared using a *t* test, whereas sex, grade, and muscle invasion were compared using a chi-square test. The reproducibility of radiomics features was assessed using the intraclass correlation coefficient (ICC 2.1) calculated using pingouin (version 0.3.8)^25^. ICC values were interpreted as poor (< 0.50), moderate (0.50–0.75), good (0.75–0.90), and excellent (> 0.90)^26^. A p-value less than 0.05 was regarded as statistically significant.

## Results

### Clinical characteristics

The clinical characteristics of the patients assigned to the training and test datasets are shown in Table 1. The patient ages ranged from 47 to 94 years (mean, 73.6 years). There was no statistically significant difference regarding age, sex, histological grade, or muscle invasion between the training and test datasets.

**Table 1:** Patient characteristics.

	Training set	Test set	p-value
Number of patients	140	30	
Age (years, mean ± SD)	73.5 ± 8.8	73.9 ± 10.3	0.84
Sex			0.62
Male	113	23	
Female	27	7	
Histological grade			0.97
High	112	24	
Low	24	6	
Others	4	0	
Muscle invasion			0.98
MIBC	51	11	
NMIBC	89	19	

*SD* standard deviation, *MIBC* muscle-invasive bladder cancer, *NMIBC* non-muscle-invasive bladder cancer.

### Segmentation performance in the training and test datasets

The mean DSCs with different sequences as input data for five-fold cross-validation are shown in Table 2. Our U-Net model with multi-sequence images (b0 images, b1000 images, and ADC maps for triple channels) achieved the highest mean DSC of 0.83 and 0.79 in the training and validation datasets, respectively. Consequently, we decided to adopt multi-sequence images as the input data for the final model. The mean DSCs for the validation dataset of five-fold cross-validation with multiple-sequence images using original U-Net, attention U-Net, UNet++, U^2^-Net, and TransUNet were 0.75, 0.74, 0.46, 0.69, and 0.75, respectively. These DSCs were lower than those for our modified U-Net model.

**Table 2:** Dice similarity coefficient with different sequences as input data for cross-validation.

	Training	Validation
b0	0.69 (0.64–0.75)	0.37 (0.29–0.46)
b1000	0.79 (0.77–0.80)	0.64 (0.37–0.75)
ADC	0.78 (0.76–0.81)	0.66 (0.59–0.71)
b0 + b1000 + ADC	0.83 (0.81–0.84)	0.79 (0.78–0.81)

Data are presented as the mean and range of five cross-validation models.

*ADC* apparent diffusion coefficient map, *DSC* dice similarity coefficient.

**Figure 4:**
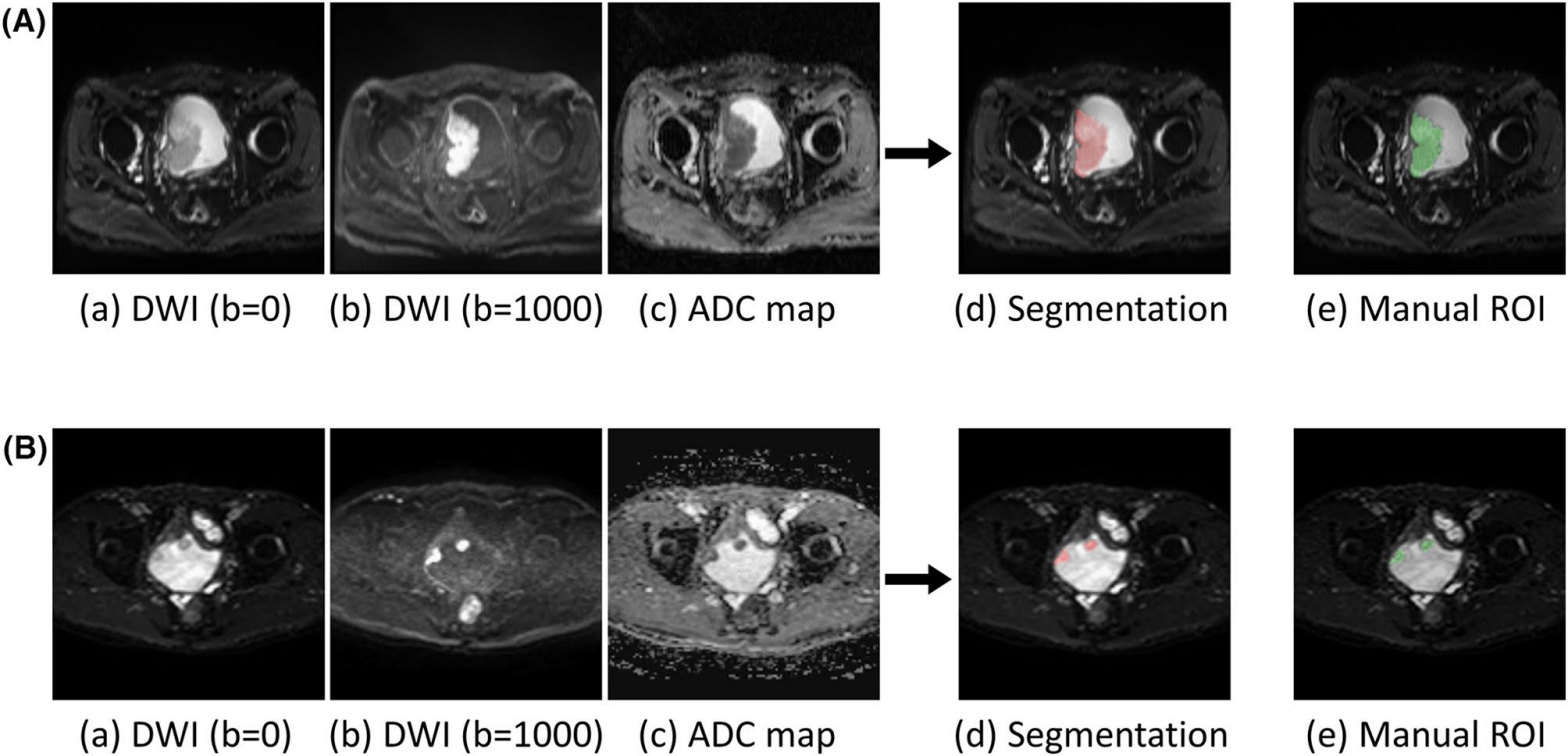
Two representative cases of automatic segmentation of bladder cancer in the test dataset (case 1 (**A**); case 2 (**B**)). (**a**) Diffusion weighted image with b = 0 s/mm^2^, (**b**) diffusion weighted image with b = 1000 s/mm^2^, (**c**) apparent diffusion coefficient map, (**d**) results of automatic segmentation overlayed on diffusion weighted image with b = 0 s/mm^2^, (**e**) manual region of interest for the reference standard. The large tumor was almost perfectly segmented in case 1 (Dice similarity coefficient = 0.95), and the two distant tumors were well segmented in case 2 (Dice similarity coefficient = 0.82).

The median DSC of the final model with multi-sequence images for the test dataset was 0.81 (interquartile range, 0.70–0.88). Representative cases of automatic segmentation for the test dataset are presented in Fig. 4.

### Reproducibility of radiomics features

**Figure 5:**
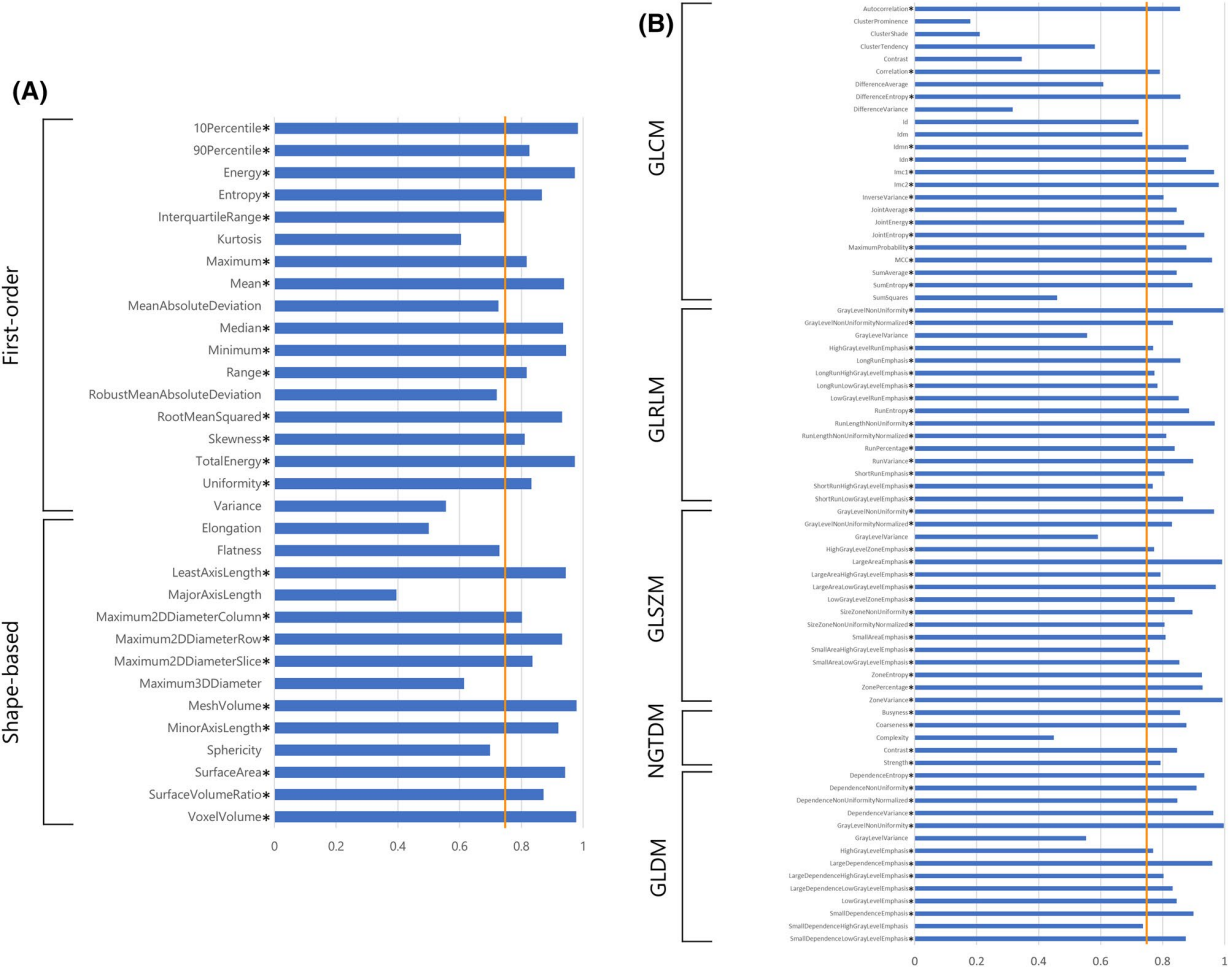
Intraclass correlation coefficient (ICC) values for radiomics features obtained by manual and automatic segmentation (first-order and shape-based features (**A**); high-order features (**B**)). Asterisks indicate the features with good to excellent ICC values (ICC > 0.75; orange line). *GLCM* gray-level co-occurrence matrix, *GLRLM* gray-level run length matrix, *GLSZM* gray-level size zone matrix, *NGTDM* neighboring gray tone difference matrix, *GLDM* gray-level dependence matrix.

The ICC values of the radiomics features derived from manually and automatically segmented BC are presented in Fig. 5. Good to excellent reproducibility was confirmed (ICC, 0.75–0.98) regarding first-order and shape-based features except for kurtosis, mean absolute deviation, robust mean absolute deviation, variance, elongation, flatness, major axis length, maximum 3D diameter, and sphericity (ICC, 0.39–0.73). Among the higher-order features, 61/75 features demonstrated good to excellent reproducibility (ICC, 0.76–1.00). All features with good to excellent ICC returned a significant p-value < 0.05 for the ICC analysis. The ICC values for each feature group are summarized in Table 3. All of the median ICCs of the radiomics feature groups showed good reliability (ICC, 0.83–0.86).

**Table 3:** Median and interquartile range for the intraclass correlation coefficient (ICC) values per feature group.

Features group	Number of features	Median ICC	Interquartile range
First-order features	18	0.83	0.77–0.94
Shape-based features	14	0.85	0.71–0.94
GLCM	24	0.85	0.60–0.88
GLRLM	16	0.84	0.78–0.87
GLSZM	16	0.85	0.80–0.94
NGTDM	5	0.85	0.79–0.86
GLDM	14	0.86	0.81–0.93

*GLCM* gray-level co-occurrence matrix, *GLRLM* gray-level run length matrix, *GLSZM* gray-level size zone matrix, *NGTDM* neighboring gray tone difference matrix, *GLDM* gray-level dependence matrix.

## Discussion

In this two-center study, we developed the automatic segmentation model of BC on multi-vendor MR images using a CNN. The model with multi-sequence MR images exhibited higher segmentation performance compared to that of the single-sequence models. Our final model demonstrated high segmentation performance with a median DSC of 0.81 for the test dataset. Radiomics features calculated from the automatic segmentation results by our model showed high reproducibility in terms of first-order, shape-based, and higher-order features.

Several single-center studies have performed automatic segmentation of BC, and some of these studies reported DSCs higher than 0.8^15–17^. However, machine learning studies conducted at a single site on a small number of cases carry the risk of generalization performance degradation due to overfitting^27^. To resolve this problem, we conducted the first study of automatic segmentation of BC with multi-vendor MR scanners at two institutes with the largest number of cases to date. This study design increased the generalization performance of our segmentation model because it handled MR images of diverse cohorts of patients with BC using diverse MR scanners and parameters. Despite the heterogeneity of images collected in our study, our U-net model achieved a median DSC of 0.81 for the test dataset. In addition, our model outperformed previously reported segmentation models, including the original U-Net. These results suggest that our U-net model can be applied to clinical practice and large-scale research using a radiomics approach.

This study also reported the effects of differences in input images on segmentation performance. Our U-Net model with multi-sequence MR images exhibited superior performance compared to that with single-sequence images. A similar study has been conducted on cervical cancer^28^. In accordance with our findings, the model with multi-sequence images of b0 images, b1000 images, and ADC maps exhibited the highest DSC of 0.82.

Most of the radiomics features derived from manually and automatically segmented BC exhibited good to excellent reproducibility for first-order, shape-based, and high-order features. To date, the robustness of radiomics features of BC has not been reported, but several studies on cervical and endometrial cancers exist^28,29^. A previous study on uterine cervical cancer reported poor reliability of features derived from manually and automatically segmented tumor except for first-order features^28^. A study on uterine endometrial cancer presented good to excellent reliability for many of the features but poor reliability for GLCM, GLRLM, and NGTDM^29^. Although direct comparison of BC results with those of other organs is difficult, radiomics features obtained from our U-Net model yielded better reliability compared to those in previous reports. Accordingly, our U-Net model may facilitate radiomics studies with a large number of BC cases.

There were several limitations in this study. First, we focused on DWIs and did not deal with T2WIs or dynamic contrast-enhanced T1WIs. One reason for this was bladder deformation associated with fluctuating urine volume in the bladder during scanning. Although using other sequences including T2WIs as input data with deformable image registration may have improved segmentation performance, there was a risk of misregistration leading to diminished accuracy of automatic segmentation. Our U-Net-based model performed sufficiently accurate segmentation solely using DWIs and ADC maps possibly because b0 images partially contained the information of T2WIs. Second, the cases from the two institutes were mixed and divided into training and test datasets, and external validation was not performed. Validation studies including BC cases from other institutions are warranted to demonstrate the robustness of our segmentation model. Third, the sample size of the test dataset might be small for evaluating ICC with sufficient statistical power. However, this was unavoidable, given the overall number of patients included in this study.

In conclusion, we developed a U-Net model that could accurately segment BC on MRI obtained with two-center, multi-vendor scanners. First-order, shape-based, and higher-order radiomics features extracted from the automatic segmentation results exhibited high reproducibility.

## Supplementary Information

Supplementary Information.

## Data Availability

The datasets generated during and/or analysed during the current study are available from the corresponding author on reasonable request.
