# Supplementary material for: Automatic segmentation of bladder cancer on MRI using a convolutional neural network and reproducibility of radiomics features: a two-center study
Source: Sci Rep. 2023 Jan 12;13:628. doi: 10.1038/s41598-023-27883-y (PMC9837183; doi:10.1038/s41598-023-27883-y)
Supplement: Supplementary file 1 — Supplementary Information. [file 41598_2023_27883_MOESM1_ESM.docx]

Supplementary Table. MR scanners and DWI parameters of the two institutes.

|  | KUHP |  |  |  | ORC |  |  |  |
| --- | --- | --- | --- | --- | --- | --- | --- | --- |
| Scanner | Skyra | Prisma | Avanto |  | Achieva | Ingenia | Intera | SIGNA EXCITE |
| Vendor | Siemens | Siemens | Siemens |  | Philips | Philips | Philips | GE |
| Patients | 61 | 19 | 4 |  | 31 | 26 | 27 | 2 |
| Magnetic field strength (T) | 3.0 | 3.0 | 1.5 |  | 3.0 | 3.0 | 1.5 | 1.5 |
| b-value (s/mm^2^) | 0, 100, 500, 1000 | 0, 100, 500, 1000 | 0, 500, 1000 |  | 0, 1000 | 0, 1000 | 0, 1000 | 0, 1000 |
| TR (ms) | 3200 | 3200 | 3900 |  | 2600 | 4000 | 2600 | 4250 |
| TE (ms) | 71 | 46 | 93 |  | 62 | 67 | 71 | 70 |
| Slice thickness (mm) | 4 | 4 | 5 |  | 4 | 4 | 3 | 5 |
| Gap (mm) | 1 | 1 | 1 |  | 0.4 | 0.4 | 0.3 | 0 |
| Matrix | 128×100 | 128×100 | 128×96 |  | 110×80 | 128×112 | 110×80 | 192×128 |
| FOV (mm) | 320 × 250 | 320 × 250 | 320 × 320 |  | 300 × 300 | 350 × 350 | 300 × 300 | 320 × 250 |

KUHP, Kyoto University Hospital; ORC, Osaka Red Cross Hospital; TR, repetition time; TE, echo time; FOV, field of view.
